# Supplementary material for: Gametocyte-specific and all-blood-stage transmission-blocking chemotypes discovered from high throughput screening on Plasmodium falciparum gametocytes
Source: Commun Biol. 2022 Jun 6;5:547. doi: 10.1038/s42003-022-03510-w (PMC9170688; doi:10.1038/s42003-022-03510-w)
Supplement: Supplementary file 2 — Supplementary Information [file 42003_2022_3510_MOESM2_ESM.pdf]

Supplementary information

Gametocyte-specific and all-blood-stage transmission-blocking chemotypes discovered from high throughput screening on *Plasmodium falciparum* gametocytes

Authors

Giacomo Paonessa[1*], Giulia Siciliano[2*], Rita Graziani[1], Cristiana Lalli[1], Ottavia Cecchetti[1], Cristina Alli[1], Roberto La Valle[2], Alessia Petrocchi[3], Alessio Sferrazza[3], Monica Bisbocci[1], Mario Falchi[4], Carlo Toniatti[1,3], Alberto Bresciani[1#], Pietro Alano[2#]

Affiliations

[1] Department of Translational and Discovery Research, IRBM S.p.A., Pomezia (Roma), Italy.
[2] Dipartimento di Malattie Infettive, Istituto Superiore di Sanità, Roma, Italy.
[3] Department of Drug Discovery, IRBM S.p.A., Pomezia (Roma), Italy.
[4] Centro Nazionale AIDS, Istituto Superiore di Sanità, Roma, Italy.

[*] These authors contributed equally
[#] Corresponding authors

Corresponding authors

Alberto Bresciani, Department of Translational and Discovery Research, IRBM S.p.A., Via Pontina Km 30,600, 00071 Pomezia (Roma), Italy. E-mail: a.bresciani@irbm.com

Pietro Alano, Dipartimento di Malattie Infettive, Istituto Superiore di Sanità, Viale Regina Elena 299, 00161 Roma, Italy. E-mail: pietro.alano@iss.it

Supplementary Figure 1

Production of the *P. falciparum* NF54 *hsp86*-PpyRE13 parasite line

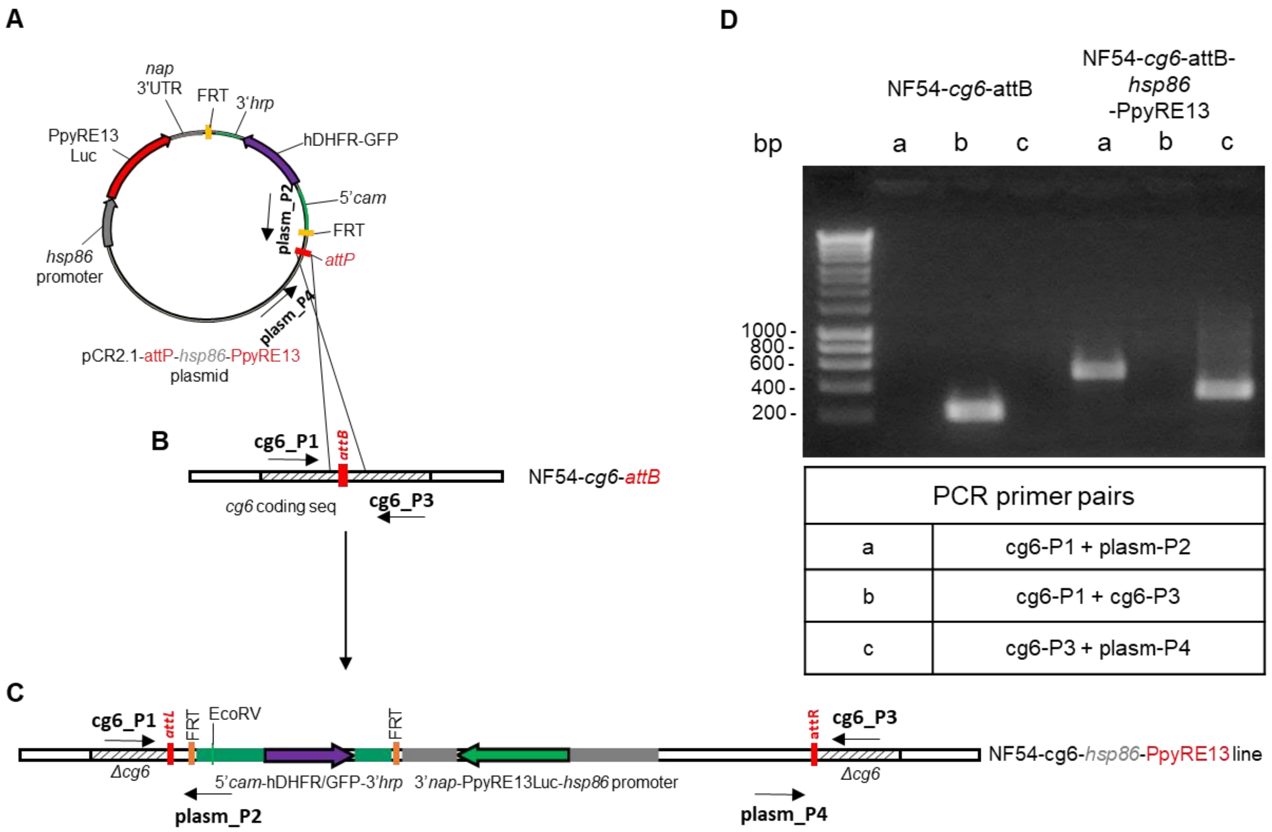

(**A**) Map of the pCR2.1-*attP-hsp86*-PpyRE13 plasmid. The *Photinus pyralis* PpyRE13 luciferase gene is flanked by the *P. falciparum hsp86* promoter and the *nap* 3' UTR. The plasmid contains an *attP* site for integration into a parasite *attB* site (Nkrumah et al, 2006). (**B**) Map of the NF54-*cg6-attB* locus. The *cg6* gene is interrupted by an *attB* site. (**C**) Structure of the *hsp86*-PpYRE13 luciferase cassette integrated in the *cg6* gene of the NF54-cg6-*attB P. falciparum* line. (**D**) PCR analysis of the genomic DNA extracted from parasites of the parental NF54-*cg6-attB* line and recombinant NF54 *hsp86*-PpyRE13 strain.

Supplementary Figure 2

Identity and purity of the novel chemotypes

(4-((1H-benzo[d]imidazol-1-yl)methyl)piperidin-1-yl)(5-nitrofuran-2-yl)methanone, **69**

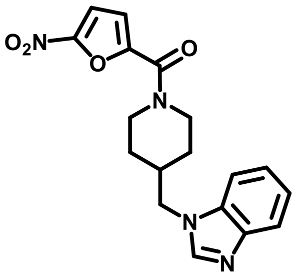

^{1}H NMR (400 MHz, DMSO-d_6) δ ppm 8.29 (s, 1H), 7.75 (d, *J*=3.7 Hz, 1H), 7.68 (t, *J*=8.7 Hz, 2H), 7.31 - 7.20 (m, 3H), 4.37 (m, 1 H), 4.22 (d, *J*=7.0 Hz, 2H), 4.12 (m, 1H), 3.16 (m, 1H), 2.79 (m, 1H), 2.22 (m, 1H), 1.58 (m, 2H), 1.29 (m, 2H); ^{13}C NMR (125 MHz, DMSO-d_6): δ ppm 161.2, 156.6, 147.9, 144.2, 142.7, 141.6, 122.4, 121.6, 119.2, 116.5, 112.8, 110.7, 48.9, 36.0; HRMS (m/z): [M]+ calcd. for $C_{18}H_{18}N_4O_4$, 355.1401; found, 355.1399.

(propane-1,3-diylbis(piperidine-1,4-diyl))bis((4-chlorophenyl)methanol), **13**

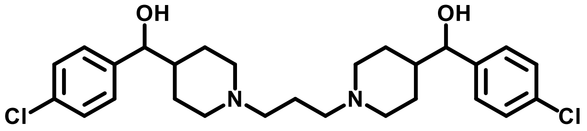

^{1}H NMR (400 MHz, DMSO-d_6) δ ppm 10.22 (brs, 2H), 7.40 (d, *J*=8.3 Hz, 4H), 7.33 (d, *J*=8.4 Hz, 4H), 5.53 (s, 2H), 4.35 (s, 2H), 3.50 - 3.35 (m, 3H), 3.20 - 3.17 (m, 1H), 3.12 - 3.03 (m, 4H), 2.81 - 2.76 (m, 4H), 2.13 (m, 2H), 1.88 - 1.85 (m, 2H), 1.71 (m, 2H), 1.63 - 1.57 (m, 4H). 1.46 - 1.43 (m, 2H); ^{13}C NMR (125 MHz, DMSO-d_6): δ ppm 142.8, 131.4, 128.3, 127.9, 74.5, 52.9, 51.5, 40.4, 25.4, 24.5; HRMS (m/z): [M]+ calcd. for $C_{27}H_{36}Cl_2N_2O_2$, 491.2227; found, 491.2226.

Supplementary Figure 2 (cont.)

2-(((6-methyl-2-phenylimidazo[1,2-a]pyridin-3-yl)methyl)thio)pyridine 1-oxide, **16**

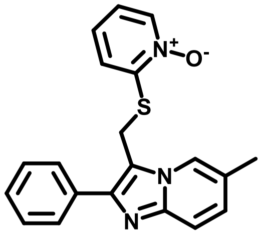

^{1}H NMR (400 MHz, DMSO-d_6) δ ppm 8.43 (s, 1H), 8.35 (d, *J*=3.9 Hz, 1H), 7.85 (d, *J*=8.2 Hz, 2H), 7.60 - 7.57 (m, 2H), 7.51 (t, *J*=8.7 Hz, 2H), 7.42 - 7.30 (m, 2H), 7.28 - 7.23 (m, 2H), 4.82 (s, 2H), 2.35 (s, 3H)); ^{13}C NMR (125 MHz, DMSO-d_6): δ ppm 153.7, 149.6, 143.6, 138.2, 134.1, 128.8, 128.3, 127.8, 127.7, 125.3, 123.3, 122.5, 121.9, 121.7, 116.3, 112.6, 24.7, 17.7; HRMS (m/z): [M]+ calcd. for $C_{20}H_{17}N_3OS$, 348.1165; found, 348.1162.

Supplementary Table 1

Oligonucleotide sequences

Cg6_P1: GAAAATATTATTACAAAGGGTGAGG
Cg6_P3: CTCTTCTACTCTTTCGAATTC
Plasm_P2: GGTTAACGAAGTTCCTATACTTTCTAG
Plasm_P4: GACTGGAAAGCGGGCAGTGAG
