# Supplementary material for: Gametocyte-specific and all-blood-stage transmission-blocking chemotypes discovered from high throughput screening on Plasmodium falciparum gametocytes
Source: Commun Biol. 2022 Jun 6;5:547. doi: 10.1038/s42003-022-03510-w (PMC9170688; doi:10.1038/s42003-022-03510-w)
Supplement: Supplementary file 3 — Description of Additional Supplementary Files [file 42003_2022_3510_MOESM3_ESM.pdf]

Description of Additional Supplementary Files

File name: Supplementary Data 1
Description: Underlying source data for all graphs and charts.

File name: Supplementary Data 2
Description: Excel file reporting activity of the 84 hits in cell assays.
